# High-Flow Nasal Cannula Oxygen Therapy: Physiological Mechanisms and Clinical Applications in Children

**DOI:** 10.3389/fmed.2022.920549

**Published:** 2022-06-03

**Authors:** Santi Nolasco, Sara Manti, Salvatore Leonardi, Carlo Vancheri, Lucia Spicuzza

**Affiliations:** ^1^Department of Clinical and Experimental Medicine, University of Catania, Catania, Italy; ^2^Pediatric Pulmonology Unit, Department of Clinical and Experimental Medicine, University of Catania, Catania, Italy

**Keywords:** high-flow nasal cannula (HFNC), oxygen therapy, children, pediatric, respiratory failure, bronchiolitis, respiratory distress

## Abstract

High-flow nasal cannula (HFNC) oxygen therapy has rapidly become a popular modality of respiratory support in pediatric care. This is undoubtedly due to its ease of use and safety, which allows it to be used in a wide variety of settings, ranging from pediatric intensive care to patients' homes. HFNC devices make it possible to regulate gas flow and temperature, as well as allowing some nebulized drugs to be administered, features very useful in children, in which the balance between therapeutic effectiveness and adherence to treatment is pivotal. Although the physiological effects of HFNC are still under investigation, their mechanisms of action include delivery of fixed concentration of oxygen, generation of positive end-expiratory pressure, reduction of the work of breathing and clearance of the nasopharyngeal dead space, while providing optimal gas conditioning. Nevertheless, current evidence supports the use of HFNC mainly in moderate-to-severe bronchiolitis, whereas for asthma exacerbations and breath sleeping disorders there is a lack of randomized controlled trials comparing HFNC to continuous positive airway pressure (CPAP) and non-invasive ventilation (NIV), which are essentials for the identification of response and non-response predictors. In this regard, the development of clinical guidelines for HFNC, including flow settings, indications, and contraindications is urgently needed.

## Introduction

High-flow nasal cannula (HFNC) oxygen therapy was first introduced into clinical practice in the early 2000s as an alternative to continuous positive airway pressure (CPAP) to manage apnea in premature neonates (1). Since then, its use in infants and children with respiratory failure has steadily grown. Indeed, nowadays HFNC is an extremely popular mode of respiratory support in pediatric care, due to a number of factors including the availability of easy-to-use devices that are exceptionally well tolerated by most of the patients, as compared to CPAP or other modes of non-invasive ventilation (NIV) (2). The HFNC apparatus is designed to provide heated and humidified gasses, usually air mixed with oxygen, at different flow rates and adjustable concentrations. The gas is inhaled *via* a soft and comfortable silicone nasal cannula that fits without occluding the nose. Although the term “high flow” is generally opposed to “low flow” used for conventional oxygen therapy (COT), there is no precise definition of what constitutes a high flow, as rates vary according to the age and weight of the patient, ranging from 2 to 60 L/min (3, 4).

While originally limited to pediatric intensive care units (PICU), because of its ease of use, HFNC has now expanded to a variety of settings, including emergency departments, inpatient pediatric wards and even patients' homes (4, 5). As a result, the body of literature on pediatric HFNC, despite still being scant compared to the broader range of adults, has steadily grown. Due to the proven safety and beneficial effects of heated humidified high flows, the future applications of HFNC in the pediatric setting will likely increase in the coming years. Therefore, the aim of this review is to report information about the most updated understanding on the action mechanism in children, addressing relevance and limitations of the current research, in order to provide an outlook on potential future perspectives.

## Devices and Settings

**Figure 1:**
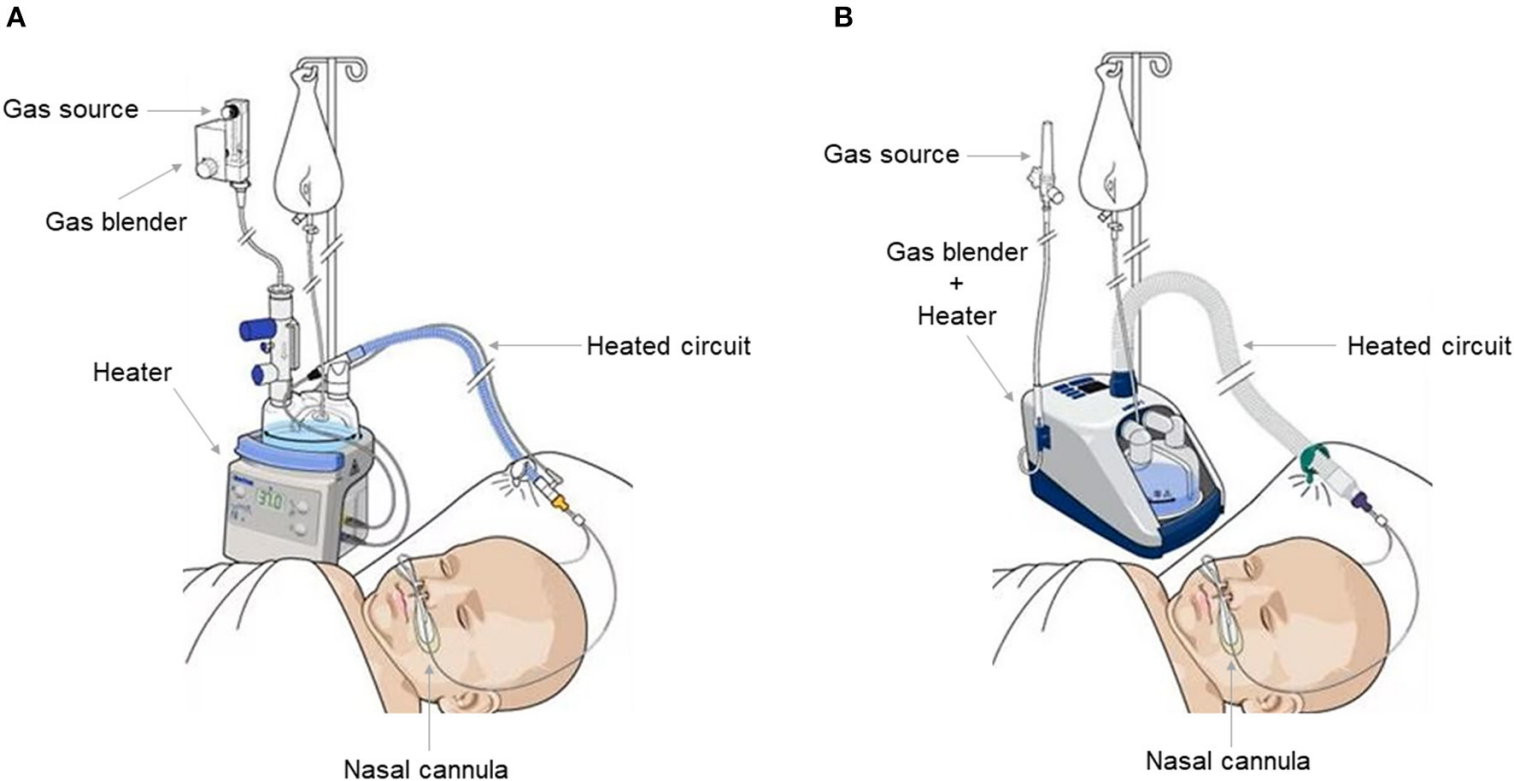
Fisher and Paykel Optiflow system ^®^ **(A)** and Fisher and Paykel Airvo 2 ^®^ system **(B)**. Both allow an inhaled oxygen fraction of up to 100% and generate a flow of up to 60 L/min. The Fisher and Paykel Airvo 2 ^®^ system combines a gas mixer and heater in one device.

Three types of HFNC devices are currently available for pediatric patients. The first type, utilized by Optiflow System^®^ (Fisher and Paykel, Auckland, New Zealand) (Figure 1A), Precision Flow^®^ (Vapotherm, Exeter, UK), and Comfort-Flo^®^ (Teleflex Medical, Durham, NC, USA) consists of an air/oxygen blender that is connected to a system to humidify and heat the gas. The device can be equipped with a pressure relief valve that cuts off the flow when a predetermined pressure in the circuit is reached. The second type, employed by Airvo2^®^ (Fisher and Paykel, Auckland, New Zealand) (Figure 1B), works through an integrated turbine generating the flow plus a heated humidifier with the advantage of not requiring an external source of gas, except from oxygen and nitric oxide. The third type is based on a CPAP or conventional ventilator with an HFNC breathing circuit connected to the humidifier.

There is no universal consensus among pediatricians about the optimal flow. As such, information about appropriate settings has been retrieved from the most relevant clinical studies in acute bronchiolitis (6–8). Patients younger than 24 months could tolerate a flow of 1–2 L/kg/min (up to 20 L/min). Superior flows had the same reported efficacy but resulted uncomfortable (8). Considering the child weight:

- 1–2 L/kg/min are recommended up to 10 kg;
- 1 L/kg/min from 10 up to 20 kg;
- 0.8–1 L/kg/min from 20 up to 40 Kg;
- 0.5–1.1 L/kg/min for >40 Kg.

Cannula size should also be chosen according to age and body weight. The cross-sectional area of the cannula should not exceed 50% of the nostrils because of the risk of unexpected increases in airway pressure and subsequent risk of air leakage.

## Drugs Nebulization

Aerosolized drug delivery using HFNC is an attractive modality of administration since traditional nebulizer masks are often poorly tolerated by children (9). However, controversies emerged after some *in vitro* feasibility evaluation.

The HFNC presents two main limitations: (1) The aerosol administration *via* nasal cannulas increases the upper airways deposition in comparison to oral inhalation (10, 11); (2) High gas flow rate increases particle deposition by impaction (12–14). These researches suggest that aerosol particle distribution is only feasible at flows <6 L/min (9). Using vibrating mesh nebulizers placed immediately upstream or downstream of the humidification chamber, about 1–10% of the drug may be delivered to the lungs, a quantity significantly lower than amounts delivered with conventional interfaces (up to 25%) (15–17), but still sufficient to exert clinical effects in pediatric patients. To date, researches performed *in vivo* about drug nebulization using HFNC system are still few. In these studies, performed in children with respiratory distress due to asthma exacerbation or bronchiolitis, the inhalation of a bronchodilator through a nebulizer placed within the HFNC circuit was as effective as the inhalation through conventional devices, but the level of comfort was greater (18–20). The positive results observed with bronchodilators may not be generalized to other drugs (21). For example, the current HFNC devices are likely to be inefficient for antibiotics, wherein the drug volume deposited into the lung is an important factor of efficacy.

## Physiological Effects of HFNC

Studies are still ongoing to unravel the full spectrum of HFNC's mechanisms of action. It should be noted that HFNC has been rapidly introduced by clinicians into their daily practice, although some physiological aspects still remain to be clarified. A list of these effects in children is provided in Table 1.

**Table 1:** Physiological effects of COT, HFNC and CPAP/NIV.

	**Conventional oxygen therapy (COT)**	**High-flow nasal cannula (HFNC)**	**Continuous positive airway pressure (CPAP) / Non-invasive ventilation (NIV)**
Deliver fixed concentrations of oxygen and other gasses	+	+++	++
Generate positive end-expiratory pressure	=	+	+++
Reduce work of breathing	=	++	+++
Anatomic dead space washout	=	+++	+
Reduce inspiratory resistance	=	+	+++
Gas conditioning	=	+++	+
Improve mucociliary clearance	=	+++	=

*=, no effect; +, low effect; ++, medium effect; +++, high effect*.

### Delivery of Fixed Oxygen Concentrations and Other Gasses

Physiologically, inspiratory flow varies with each breath and so does the fraction of inhaled oxygen (FiO_2_) administered *via* COT at low flows (22). In patients with respiratory distress, the inspiratory flow often exceeds the oxygen flow delivered by COT, resulting in oxygen concentration dilution (23, 24). The delivery of high flows allows matching patients' inspiratory peak flow (25). As a result, the FiO_2_ reaching the lower airways is closer to the FiO_2_ delivered by the HFNC device, enabling the possibility of a fine titration of oxygen administration (26).

This feature has also been exploited to deliver nitric oxide directly to the lungs. Inhaled nitric oxide (iNO) is a selective pulmonary vasodilator that decreases pulmonary arterial pressure and pulmonary vascular resistance without inducing systemic hemodynamic effects. The iNO therapy using HFNC has been reported successfully in infants with respiratory distress and post-extubation after the Fontan procedure (27, 28).

### Generation of Positive End-Expiratory Pressure, Work of Breathing Reduction and Washout of Anatomical Dead Space

The high flows produced by HFNC devices are often sufficient to prevail against children's expiratory flow and to generate a small positive end-expiratory pressure (PEEP) in the airways (29), albeit the pressure delivered by HFNC devices to the distal airways is difficult to measure. Esophageal manometry is considered the standard for detecting pleural pressure in spontaneously breathing patients (30). This technique may be feasible in adults, but difficult to adopt in children (31). As such, measurements are often obtained in the experimental settings (29) by various indirect methods including electrical impedance tomography on the surface of the chest (32) or electrical activity of the diaphragm (33). In addition, the generation of PEEP is linearly dependent on the amount of flow and is influenced by the weight of the patient, the size of the cannula and nostrils and the degree of mouth opening (34). Therefore, all these factors combined could influence results consistency. The few studies performed measuring pressure in the pharynx and esophagus reported that a limited PEEP of 2–4 cm H_2_O was generated by HFNC in children (35, 36).

The work of breathing (WOB) is the energy expended by the respiratory muscles to perform their activity. Theoretically, lowering the respiratory rate and improving thoraco-abdominal coordination should reduce WOB. The PEEP generated by high flows has been considered one of the factors contributing to reducing WOB, *via* matching intrinsic PEEP and increasing alveolar recruitment (33, 37). This concept has been challenged by a recent study from Guglielmo and co-workers. In their trial, HFNC applied to 22 children with bronchiolitis reduced breathing effort, without a consistent increase in end-expiratory lung volume and no significant change in tidal volume or transpulmonary pressure, raising the hypothesis that PEEP application is not the primary HFNC mechanism for reducing WOB in bronchiolitis (38).

HFNC also provides a washout of the “anatomical dead space,” namely the volume of air located in the first third of the respiratory tract which does not take part in the gas exchange process. Clearance of carbon dioxide (CO_2_) from nasopharyngeal dead space can also affect the WOB, by producing a more efficient ventilation (39–41). The only *in vivo* study designed specifically to provide insight into the mechanism of dead space washout was performed on adult volunteers using tracer gas. It showed effective clearance in the upper airways directly related to the HFNC flow rate and time, with subsequently a reduction in rebreathing of expired air, which could result in improvement of alveolar ventilation and gas exchange (40). To some extent, this mechanism might contribute to the HFNC effectiveness in treating sleep breathing disorders in children. Indeed, reducing CO_2_ levels has the potential to improve breathing patterns and correct apnea and hypopnea (42). Duong et al. showed that flows delivered at 20 L/min to 4–8-year-old child airway replicas reduced end-tidal carbon dioxide from baseline values, whereas delivery of CPAP through a sealed nasal mask increased end-tidal carbon dioxide from baseline values (43). Thus, further *in vivo* studies investigating HFNC therapy as an alternative to CPAP therapy for treating OSA, should consider potential beneficial effects of improved gas washout when administering HFNC distinctly from its use to produce PEEP.

### Reduction of Inspiratory Resistance and Gas Conditioning

The nasopharynx facilitates humidification and warming of the inhaled gas by contact with its large mucosal surface area, but at the same time the passage of air through this anatomical region causes an increase in inspiratory resistance. HFNC minimizes this resistance by providing nasopharyngeal gas flows *via* nasal prongs that match or exceed a patient's peak inspiratory flow, with a positive effect on WOB (42).

Non-warmed, non-humidified air can have a detrimental impact on child upper and lower airways. First, Greenspan et al. demonstrated that respiration, even for a short time, of not warmed or humidified air resulted in a significant decrease in both pulmonary compliance and conductance in ventilated infants (44). Fontanari et al. provided a physiological explanation of this phenomenon in their study. They showed that receptors in the nasal mucosa respond to cold and dry gas to elicit a protective bronchoconstrictor response in both normal subjects (45) and asthmatics (46) mediated by the cholinergic system (47). The beneficial effects of warm, humidified air have been demonstrated *in vivo* in infants (48). In fact, although HFNC provided lower PEEP than CPAP, the pulmonary compliance was higher, corroborating the hypothesis that conditioning of respiratory gas could have an impact on the lung, particularly useful in case of asthma attack, wherein the inhalation of warm air could reduce bronchoconstriction.

### Optimization of Mucociliary Clearance

In muco-obstructive lung diseases, delivery of heated flows at core temperature and water vapor saturation may improve airway clearance. An *in vitro* study has shown that inspired gas with low humidity even for short periods could impair the function of human airway epithelial cells (49). Furthermore, air temperature is also crucial for optimal cilia movement, which occurs at 37°C (50). HFNC devices can deliver flow gasses at 100% humidity and core temperature, features particularly advantageous in hypersecretory states requiring an optimization of airway clearance, such as bronchiolitis, cystic fibrosis and bronchiectasis. At the moment, data on the effect of HFNC on such conditions are lacking. Only 2 case reports have been published so far, both showing that long-term home HFNC reduced atelectasis, hospitalization frequency and improved mucus drainage in post-acute bronchiolitis and CHARGE syndrome (51, 52).

## Clinical Applications

### Bronchiolitis

Acute bronchiolitis is one of the most frequent diseases in children under 2 years (53). Multiple microorganisms are responsible for its clinical manifestations, but respiratory syncytial virus is by far the most common (53). In recent years, the use of HFNC has progressively gained popularity over the current standard of care with COT, especially in case of moderate-to-severe acute bronchiolitis. Indeed, this nosological entity represents the main indication for HFNC in patients older than neonates.

Two large clinical trials benchmarked the HFNC vs. COT (6, 7). Both reported a lower treatment failure rate, defined as escalation of care during that hospital admission, in the HFNC group. However, they showed no differences in duration of hospital stay, duration of oxygen therapy, or PICU admission in comparison with COT. A recent systematic review by Lin et al. likened the effectiveness of HFNC, CPAP and COT in acute bronchiolitis (54). They found that HFNC and CPAP were both superior to COT, but treatment failure events were significantly more frequent in the HFNC group when compared to the CPAP group. However, the authors included patients with any degree of bronchiolitis severity, without performing a subgroup analysis in children with moderate-to-severe bronchiolitis. This limitation was addressed by Catano-Jaramillo and colleagues in their meta-analysis (55). They showed that both CPAP and HFNC reduced the risk for intubation, but a lower rate of therapeutic failures was found with CPAP, confirming the previous results also in this cluster of patients. Noteworthy, despite being superior to HFNC, CPAP produced more adverse events, such as skin lesions, and was less tolerated. Data available indicate that HFNC being superior to COT, despite inferior to CPAP, could play a role in the rescue therapy for children with moderate-to-severe bronchiolitis because of its ease of use and safety.

### Asthma

Due to its beneficial effects on the respiratory system, HFNC treatment may reduce the WOB during asthma exacerbations. Furthermore, the use of heated and humidified gas limits the bronchoconstriction induced by cold dry gas and improves airways cilia movement, contributing to mobilization of mucus plugs, hallmark of acute asthma attacks (56). To date, few reports explored the use of HFNC during asthma exacerbation. Two retrospective studies (57, 58) showed that treatment with HFNC improved heart rate, respiratory rate, SpO_2_/FiO_2_ ratio, pH level, and CO_2_ tension after 3–24 h compared to COT. These findings were confirmed by a prospective pilot trial by Ballestero et al. (59), in which 62 children (1–14 years) with moderate-to-severe asthma exacerbations were randomly assigned to HFNC or COT. Two hours after treatment, 53% of the children in the HFNC group demonstrated a decreased pulmonary score by at least 2 points vs. 28% in the COT group (*p* = 0.01). However, no between-group differences were observed in terms of PICUs admission and hospital length of stay. Pilar et al. (60) compared the efficacy of HFNC vs. NIV in a retrospective analysis. Twenty children received HFNC and eight of them escalated to NIV whilst 22 received NIV without treatment failure (*p* < 0.001). These authors suggested cautions when using HFNC over NIV, since it could potentially delay the initiation of NIV, resulting in longer periods of respiratory support and hospital stay.

### Respiratory Support in Case of Congenital Heart Diseases

It is widely known that high PEEP values impede venous return and increases central venous pressure (61). In contrast to CPAP, HFNC supports respiration generating minimal PEEP values (29), and therefore its effect on central venous pressure is negligible (42). This mechanism may be of particular interest for respiratory support in patients with delicate hemodynamic balance, in whom a high PEEP could exerts deleterious effects. In a randomized controlled trial (RCT) in pediatric patients with congenital heart disease undergoing procedural sedation, the use of HFNC compared with COT, reduced the incidence of desaturation, the need for NIV and the risk of CO_2_ retention without causing hemodynamic instability (62). A case report on a 10-years old patient with Fontan circulation showed that in comparison to COT, HFNC reduced heart rate, systemic vascular resistance, pulmonary vascular resistance, increased cardiac output and improved cerebral circulation, measured by near-infrared spectroscopy (63). These effects were likely due to optimal oxygenation achieved without an increase in central venous pressure which helped to suppress adrenergic activity. Naohiro et al. conducted a retrospective study (64) on HFNC *versus* NIV for acute respiratory failure after cardiac surgery in children with inborn cardiac defects. The reintubation rate within 28 days was significantly lower in the HFNC group (3 vs. 26%, *p* = 0.04). Furthermore, the HFNC group's PICU stays were significantly shorter than those of the NIV group (10 days vs. 17 days, *p* = 0.009).

### Obstructive Sleep Apnea

Obstructive sleep apnea (OSA) is the result of upper airway obstruction during sleep (65, 66). Children with OSA are at increased risk for neurocognitive and cardiovascular conditions (67, 68). The current treatment options for OSA in children include adenotonsillectomy, when applicable, and CPAP (69) with the latter often impeded by limited adherence (70, 71). Already in 2009 Brian McGinley and colleagues delivered high flows (20 L/min) to 12 children with mild-to-severe OSA, showing that the reduction in the apnea-hypopnea index on HFNC was comparable to that on CPAP (72). More recently, in two observational studies (73, 74) conducted in CPAP-intolerant children with moderate-to-severe OSA, HFNC reduced nocturnal respiratory events and improved oxygen saturation. In a case series (75), long-term home HFNC was successful in treating five children with severe OSA. Despite this limited evidence, HFNC might be considered as a rescue option in children not compliant to CPAP treatment. However, RCTs comparing CPAP to HFNC are warranted to provide definitive results.

### Pneumonia

The HFNC role in the management of children with acute respiratory failure due to pneumonia includes two RCTs (76, 77). In the first one (76), there was no difference in treatment failure between bubble CPAP and HFNC, but the study was stopped prematurely because of the high mortality rate in the COT group. Later, Cong Liu and co-workers (77) evaluated 84 children under 2 years of age in a RCT on HFNC vs. CPAP in the management of mild-to-moderate respiratory failure due to pneumonia. No differences were observed in terms of treatment failure necessitating intubation and transfer to the PICU, duration of hospital stay, non-invasive respiratory support and mortality. In addition, the HFNC group had a lower level of nasal injury, abdominal distension and better tolerance. However, since low PaO_2_/FiO_2_ ratio was associated with HFNC failure, the authors were cautious suggesting that HFNC should be considered as an intermediate level of respiratory support between COT and NIV.

### Future Potential Use of the HFNC

Bronchiectasis and cystic fibrosis are associated with a chronic mucus secretion. For these conditions improving the muco-ciliary clearance is pivotal in order to prevent recurrent infections and therefore to preserve long-term function. The use of HFNC is particularly promising in the management of these conditions (78) for the aforementioned beneficial effects of humidified and heated gas flows on the airway cilia. To date, no studies have been published so far on pediatric patients with cystic fibrosis and only two case reports evaluated the effectiveness of long-term home HFNC in children with bronchiectasis with reduction in the frequency of pulmonary infection (51, 52).

Interhospital transport is a delicate moment for an ill child. Clinical deterioration following interhospital transport accounts for 30% of entire PICU admission, and is associated with increased rate of invasive ventilation use and prolonged PICU stay (79). An Australian study published in 2021 (80) reported that the implementation of HFNC on interhospital transport was associated with reduced PICU length of stay and respiratory support use, thus supporting its employment in this setting.

Further case reports have also described the effects of HFNC in children with acute pulmonary edema (81) and a pediatric burn patient with post extubation stridor (82).

## Conclusion

The HFNC is a relatively safe and well-tolerated respiratory support suitable to a broad range of hospital and domiciliary settings. Several physiological mechanisms are responsible for its effectiveness. Studies published so far support their superiority over COT in almost every condition, with stronger evidence for rescue therapy for acute bronchiolitis. Notwithstanding, better designed and controlled studies are required to define the role of HFNC vs. CPAP and NIV, in order to better understand the predictors of non-response and avoid respiratory support escalation delay.

## Author Contributions

All authors made substantial, direct, and intellectual contributions to the work and approved the submitted version.

## Conflict of Interest

The authors declare that the research was conducted in the absence of any commercial or financial relationships that could be construed as a potential conflict of interest.

## Publisher's Note

All claims expressed in this article are solely those of the authors and do not necessarily represent those of their affiliated organizations, or those of the publisher, the editors and the reviewers. Any product that may be evaluated in this article, or claim that may be made by its manufacturer, is not guaranteed or endorsed by the publisher.

## Abbreviations

CO_2_: carbon dioxide
COT: conventional oxygen therapy
CPAP: continuous positive airway pressure
FiO_2_: fraction of inhaled oxygen
HFNC: high-flow nasal cannula
iNO: inhaled nitric oxide
NIV: non-invasive ventilation
OSA: obstructive sleep apnea
PEEP: positive end-expiratory pressure
PICU: pediatric intensive care unit
RCT: randomized control trial
WOB: work of breathing.
